# Association between Concentric and Eccentric Isokinetic Torque and Unilateral Countermovement Jump Variables in Professional Soccer Players

**DOI:** 10.3390/jfmk7010025

**Published:** 2022-02-25

**Authors:** Bruno Mazziotti Oliveira Alves, Robson Dias Scoz, Ricardo Lima Burigo, Isabella Christina Ferreira, Ana Paula Silveira Ramos, Jose Joao Baltazar Mendes, Luciano Maia Alves Ferreira, Cesar Ferreira Amorim

**Affiliations:** 1 Masters and Doctoral Program in Physiotherapy, University City of Sao Paulo (Unicid), Sao Paulo 03071-000, Brazil; brunomazziotti@hotmail.com (B.M.O.A.); ricardoburigo@gmail.com (R.L.B.); isabellachristinaf@hotmail.com (I.C.F.); 2 Department of Physical Therapy and Biomechanics, Arsenal Football Club, London N7-7AJ, UK; 3 Physiotherapy Department, University of South of Santa Catarina (Unisul), Sao Jose 88137-270, Brazil; ana.p.ramos@animaeducacao.com.br; 4 Laboratory of Physical and Functional Assessment (LAFF), Interdisciplinary Center of Investigation Egas Monis (CiiEM), Caparica, 2829-511 Setubal, Portugal; jmendes@egasmoniz.edu.pt (J.J.B.M.); lucianomaia@egasmoniz.edu.pt (L.M.A.F.); 5 Lab Corinthians R9, Sport Club Corinthians Paulista, Sao Paulo 03828-000, Brazil; 6 Laboratoire de Recherche BioNR, Université du Quebec, Saguenay, QC G7H-2B1, Canada

**Keywords:** biomechanics, performance analysis, vertical jump

## Abstract

Isokinetic tests have been highly valuable to athletic analysis, but their cost and technical operation turn them inaccessible. The purpose of this study was to verify the correlation between unilateral countermovement jump variables and isokinetic data. Thirty-two male professional soccer players were subjected to the isokinetic testing of both knee extensors and flexors in concentric and eccentric muscle contractions. They also executed unilateral countermovement vertical jumps (UCMJ) to compare maximum height, ground reaction force, and impulse power with isokinetic peak torque. Data analysis was conducted through Pearson correlation and linear regression. A high correlation was found between dominant unilateral extensor concentric peak torque and the UCMJ maximum height of the dominant leg. The non-dominant leg jump showed a moderate correlation. No other variable showed statistical significance. Linear regression allowed the generation of two formulae to estimate the peak torque from UCMJ for dominant and non-dominant legs. Although few studies were found to compare our results, leading to more studies being needed, a better understanding of the unilateral countermovement jump may be used in the future as a substitute to the expensive and technically demanding isokinetic testing when it is unavailable, allowing the assessment of lower limb physical asymmetries in athletic or rehabilitation environments.

## 1. Introduction

Soccer is considered a high-intensity, intermittent team sport that requires the development of multiple athletic qualities including sprinting, jumping, and changing of direction [1,2]. These physical demands generate high levels of injury risk on lower limb muscles [3].

Isokinetic tests have been highly valuable for muscle analysis and injury prevention, have been extensively used in professional soccer for over 20 years, and are considered by coaches, trainers, physical therapists, and sports physicians as an important instrument for player performance measurement. There are thousands of scientific publications on a variety of applications, such as injury risk reduction, training regime follow-up, and lower limb strength symmetry assessment [4,5]. However, the cost and technical operation turn it inaccessible to most soccer teams, sports clinics, and associations.

Soccer requires jumps and locomotion with maximal acceleration over short distances, and many sports professionals have used the countermovement jump (CMJ) as a method of monitoring performance change and neuromuscular readiness in soccer athletes [6,7,8]. However, it is uncertain whether measuring jump variables can provide similar information regarding muscle function and athletic performance when compared to isokinetic assessment.

Therefore, the aim of the present study was to verify the correlation between unilateral countermovement jump variables with isokinetic data. Our hypothesis is that there is a correlation between unilateral countermovement jump variables and the isokinetic peak torque of knee extensors.

As isokinetic testing measures each leg separately, the unilateral jump could have some degree of correlation with other testing methods and a mathematical process could estimate isokinetic values from a unilateral jump. This hypothesis has emerged as an increasing number of athletic characteristics have been correlated under different unilateral testing [9,10,11,12,13].

## 2. Materials and Methods

### 2.1. Design

This is a cross-sectional study from a series of transversal data collection during the Brazilian pre-season soccer championship when all players are subjected to physical tests prior to competition. This research followed the recommendations of the Strengthening the Reporting of Observational Studies in Epidemiology (STROBE) statement [14] and its design followed the recommendations of the Improving Healthcare Decisions Task Force (ISPOR Database Recommendations) [15]. The study was carried out according to the Declaration of Helsinki, following the Ethical Standards in Sports and Exercise Science Research [16]. A protocol was fully approved by the Human Research Ethics Committee of our university with number #3652668. There was no involvement from patients or members of the public in the design, conduct, reporting, or dissemination plans of the research.

### 2.2. Participants

The participant sample size followed the example of 20–30 subjects used in a similar study with isokinetic and unilateral countermovement jump comparisons [17]. To reduce heterogenicity we limited our sample to players from the same soccer team with similar training regimes and nutritional intake. It included 32 right-side dominant, elite professional soccer players (12 midfielders, 6 strikers/forwards, 6 defenders, 4 goalkeepers, 4 backsides) who had been playing for at least five years on first and second Brazilian divisions, training regularly one to two sessions per day, six times per week.

In order to be included in this study, players had to be able to fully participate in team training sessions and match play at the time of the analysis. Players who had third-degree hamstring or quadricep muscular injury in the past 3 months, knee surgery in the past 12 months, or were currently in treatment for other muscular injuries or illnesses were excluded from our sample.

The purpose, experimental procedures, possible risks, and benefits of the study were explained to the athletes, who provided a written informed consent form to confirm participation in the study. Demographic and anthropometric information on the sample is presented in Table 1.

### 2.3. Instruments

For isokinetic data collection, we used an isokinetic dynamometer (Cybex-CSMI, model HumacNorm 2009, Stoughton, MA, USA) with a signal acquisition rate of 500 Hz. To improve players’ test understanding, we used a modified 10-points Borg scale (BORG) [18] for perceived exertion—where zero was no strength effort and 10 the maximum strength effort possible—and a visual analog pain scale, where zero was “no pain” and 10 was “worst pain imaginable” (VAS) [19].

For the countermovement analysis, we used a uniaxial force plate (Biomec 400 v1.1^®^, EMG System Ltd.a^®^), consisting of four load cells with an internal circuit that changes in electrical resistance upon the application of force (dimensions: 600 mm × 400 mm; sampling frequency: 1000 Hz, A/D converter with 16 bits of resolution).

For data storage and processing was used a MacBook Pro Notebook (Cupertino, CA, USA) equipped with Microsoft Office software package for Mac (version 2011, Redmond, Washington, DC, USA) and Statistical Package for Social Sciences (SPSS) from IBM (Armonk, NY, USA).

### 2.4. Procedures

Participants were requested to eat according to their team’s nutritionist-prescribed diet in the 48 h preceding the assessment and then refrain from eating and drinking substances other than water for one hour before the session. All tests were carried out in January, a few weeks before Brazil’s Regional Championship season starts. Players were also instructed to refrain from strenuous activities 48 h before testing.

Upon arrival, participants were provided with appropriate explanations and demonstrations of all procedures. Anthropometric data were recorded before the session began, following the International Society for the Advancement of Kineanthropometry (ISAK) Level 01 certified anthropometrist protocol.

After an interview and physical assessment, all subjects were submitted to a testing protocol following the guidelines of the APTA (American Physical Therapy Association) and soccer-specific studies using isokinetic machines [20,21]. The same physiotherapist, with 10 years of experience, performed all isokinetic tests. All isokinetic tests were performed on both legs separately to compare their results with unilateral jump variables. The testing order was the same for all subjects. Initially, the vertical unilateral countermovement jump (UCMJ) test was performed, and after 5 min of rest, the isokinetic test was administered.

To execute the concentric isokinetic test, participants were positioned on the seat of the isokinetic dynamometer and executed 10 repetitions of concentric knee flexion and extension, both with a velocity of 90 degrees per second and a range of motion of 100 degrees, for warming-up purposes (Borg up to 5, VAS up to 1, or test interrupted), followed by a rest period of 120 s. The warm-up on the isokinetic machine was chosen to improve specificity and familiarization with the following test. The athlete then performed five concentric repetitions of knee flexion and extension at 60 degrees per second for familiarization with the exercise velocity, followed by another rest period of 120 s. Then, they performed three concentric repetitions of knee flexion and extension (velocity: 60 degrees per second and range of motion: 100 degrees) with maximum effort (Borg 10), while continuously receiving the standardized verbal encouragement: “Faster”. The presence of pain equal or superior to 05 on VAS interrupted the test, canceling it and excluding the subject from the sample. The repetition with a higher peak torque value of all three repetitions was used for statistical analysis.

To execute the eccentric isokinetic test, participants performed at 60 degrees per second and a range of knee motion of 100 degrees. The subject executed 5 repetitions of warm-up and familiarization, followed by 3 repetitions at maximum effort (Borg 10), while constantly receiving the standardized verbal encouragement: “Hold it.” The presence of pain equal or superior to 05 on VAS interrupted the test, canceling it and excluding the subject from the sample. Between each set of exercises, subjects had 120 s to rest. Between each limb’s test, participants had 120 s to rest. The peak torque was extracted from the isokinetic machine by its manufacturer’s dedicated software (HumacNorm 2009, CSMi Inc., Boston, MA, USA) and normalized by each subject’s body weight.

The unilateral countermovement jumps (UCMJ) were executed after 5 min of warm-up exercise, consisting of jogging with self-paced moderate velocity and 3 submaximal UCMJs. The subjects performed three UCMJs on the force platform, with a recovery interval of 60 s after each trial. The BIOMEC400 platform’s EMGLab2 software (Biomec 400 v1.1^®^, EMG System do Brasil Ltd.a^®^) was used to obtain the vertical force of the ground reaction. All force signals recorded by the platform were filtered with a 35 Hz second-order low bandpass filter (Butterworth filter) to eliminate electronic noise.

To execute each UCMJ, players stood on the test leg in the center of the force platform with their hands on their hips and the knee of the non-jumping leg slightly flexed, so that the hovering foot was positioned at approximately the mid-shin height of the jumping leg. Players performed a countermovement to a self-selected depth, before jumping as high as possible and landing on the same test leg. No swinging of the non-jumping leg or arms was allowed. The jump height of the highest attempt was recorded in centimeters (cm). Sixty seconds of rest was provided between all trials. The players were instructed to maintain balance with the testing leg after landing for at least 3 s. If players touched the ground with the hovering leg or their hands came off their hips, the jump was deemed void and retaken after 60 s of rest.

### 2.5. Data Analysis

Demographic data extracted were age, height, weight, and handedness. Table 1 shows all demographic and anthropometric data of the sample. The descriptive analysis of the isokinetic and UCMJ data is shown in Table 2 and was composed by means, standard variation, standard error of measurement, minimum, and maximum values.

The normality of all data was confirmed using visual inspection and the Kolmogorov-Smirnov test. The homogeneity of variance was assessed via Levene’s Test. The Pearson’s correlation was used to identify positive associations between all variables from the isokinetic test and unilateral countermovement jump. Linear correlation was conducted to better analyze variable inter-relationships and the isokinetic torque’s predictability from force platform data. Confidence Intervals (95%) are provided for all measurements. The degree of correlation was classified as Very High (higher than 0.9), High (between 0.7 and 0.9), Moderate (between 0.5 and 0.7), Low (between 0.3 and 0.5), or Very Low (between 0 and 0.3).

All data were processed using SPSS v.20 (IBM, Chicago, IL, USA) with a level of statistical significance set at alpha level *p* < 0.05.

## 3. Results

The descriptive analysis results are presented in Table 2 while the inferential analysis results are presented in Table 3 and Table 4. The Pearson correlation results, between isokinetic and force platform data, demonstrate a strong correlation (r = 0.72, *p* < 0.001) between unilateral countermovement jump height and concentric peak torque of knee extensors of the dominant leg. The same analysis found a moderate correlation (r = 0.59, *p* < 0.001) when testing concentric knee extensors of the non-dominant leg and a moderate correlation (r = 0.55, *p* < 0.001) for eccentric knee extensors of the dominant leg. This analysis also found a low correlation (r = 0.46, *p* = 0.01) between eccentric knee flexors and unilateral countermovement jump height.

The main outcomes under analysis were: Concentric Peak Torque of Dominant and Non-dominant Knee Extensors (PT.Ext.R.Con and PT.Ext.L.Con), Eccentric Peak Torque of Dominant and Non-dominant Knee Extensors (PT.Ext.R.Ecc and PT.Ext.L.Ecc), Concentric Peak Torque of Dominant and Non-dominant Knee Flexors (PT.Flx.R.Con and PT.Flx.L.Con), Eccentric Peak Torque of Dominant and Non-dominant Knee Flexors (PT.Flx.R.Ecc and PT.Flx.L.Ecc), Jump Maximum Height of the Dominant and Non-dominant Legs measured from ground reaction force (R.Jump.Height.Kinetic and L.Jump.Height.Kinetic), Ground Reaction Force of Dominant and Non-dominant Legs (R.Force and L.Force), and Ground Reaction Power of the Dominant and Non-dominant Legs (R.Power and L.Power).

The linear regression results between concentric knee extensors and unilateral countermovement jump height are presented in Table 5 and Table 6. The results show a statistically significant correlation for the dominant (*p* = 0.001) and non-dominant (*p* = 0.013) legs, confirmed by the variance overall model test F of dominant (*p* < 0.001) and non-dominant legs (*p* = 0.006). The determination coefficient (R^2^) result for dominant (0.58) and non-dominant (0.41) legs suggests that almost half of the isokinetic variable can be explained by jump height. Using these regression results, the following formulae could be used to estimate the concentric peak torque of dominant and non-dominant knee extensors, respectively: y = 7.65 + 2.97x and y = 6.57 + 2.88x. In both cases, the variable “x” is the maximum countermovement jump height measured from ground reaction force.

## 4. Discussion

The purpose of this study is to verify the correlation between unilateral countermovement jump variables and isokinetic data. Our hypothesis was the presence of a correlation between unilateral countermovement jump variables and the isokinetic peak torque of knee extensors. Our results confirm our hypothesis, showing a high degree of correlation between the UCMJ maximum jump height of the dominant leg with the extensor concentric peak torque of the same leg. Moreover, there was a moderate degree of correlation between the UCMJ maximum jump height of the non-dominant leg with the extensor concentric peak torque of the same leg. Additional analysis, through linear regression, allowed the generation of two formulae to estimate the isokinetic peak torque from the UCMJ maximum height, respectively: y = 7.65 + 2.97x (for the dominant leg) and y = 6.57 + 2.88x (for the non-dominant leg). In both cases, the variable “x” is the maximum UCMJ height in centimeters measured from the ground reaction force.

To our best knowledge, very few scientific studies have tried to estimate the isokinetic peak torque using unilateral jump data from ground reaction force parameters. The most similar study tested soccer players with isokinetic and unilateral countermovement jump parameters [22]. However, their variables under analysis were bilateral asymmetries between both legs in percentages, limiting possible comparisons with our findings. Although, they also found a correlation between concentric isokinetic torque and unilateral maximum countermovement jump height. In another study [23], the unilateral countermovement jump maximum height was used as a substitute for an isokinetic machine measurement during cruciate ligament reconstruction rehabilitation phase. So, functioning as a predictor of the effect of bilateral symmetry on physical performance and possible discharge.

The second study found also shows a correlation between the isokinetic torque of concentric knee extensors and maximum countermovement jump height [17]. However, like most studies on this field, this study used bilateral jumps, limiting possible comparisons with our findings. Additionally, they did not use a force platform, which could change the interpretation of our results, since maximum height calculations may be made with different physical parameters for each piece of equipment.

Another study measuring knee extension torque and bilateral countermovement jump data after a period of Nordic exercise training showed that both isokinetic parameters and bilateral countermovement jump maximum height improved in a similar, but proportional, degree [24]. Another similar study using Pearson’s correlation tests indicated that there was a significant positive relationship between vertical jumping height and knee extension movements [17]. A multiple regression analysis indicated that linear combinations of isokinetic torques accounted for 38% and 42% of the countermovement and squat jumping height variance, respectively. In our analysis, unilateral countermovement jump maximum height accounted for 52% and 42% of isokinetic concentric knee extensors of dominant and non-dominant legs, respectively. Whether this similar but reversed relationship is coincidental is still unknown and may be investigated in future studies.

Performance inter-limb asymmetries are used as an indicator of injury risk and training markers by almost all coaches and health professionals involved in amateur or professional soccer. These asymmetries are well known from isokinetic testing, a reliable method of performance measurement [4]. However, its high cost and technical demands bring difficulties to its use for most teams, even the professional ones. Countermovement jumps have been extensively used by coaches and trainers in soccer, due to their simplicity and high correlation with performance parameters [25]. However, unilateral countermovement jumps give the possibility to add another performance variable to trainers and health professionals without costly equipment such as isokinetic machines. This has led some researchers to expand the study of inter-limb asymmetries using unilateral countermovement jumps [9,10]. This could lead to a more accessible way to perform frequent player physical screening on a large number of individuals, faster and cheaper.

Our study is not free of limitations. Our convenient sample size, though including as many subjects as similar studies, is considered small for populational mathematical estimations, and external validity is limited.

## 5. Conclusions

Our results have confirmed our hypothesis, showing a high degree of correlation between the UCMJ maximum jump height of the dominant leg with the extensor concentric peak torque of the same leg. Moreover, there was a moderate degree of correlation between the UCMJ maximum jump height of the non-dominant leg with the extensor concentric peak torque of the same leg. Additional analysis, through linear regression, allowed the generation of two formulae to estimate isokinetic peak torque from UCMJ maximum height, respectively: y = 7.65 + 2.97x (for the dominant leg) and y = 6.57 + 2.88x (for the non-dominant leg). In both cases, the variable “x” is the maximum UCMJ height in centimeters measured from ground reaction force.

Although few studies were found to compare our results, leading to more studies being needed, a better understanding of the unilateral countermovement jump may be used in the future as a substitute for the expensive and technically demanding isokinetic testing when it is unavailable, allowing the assessment of lower limb physical asymmetries in athletic or rehabilitation environments.

## Figures and Tables

**Table 1:** Demographic data of study sample.

*n* = 32	Age (Years)	Mass (kg)	Height (cm)
Mean	27.4	77.8	180
Median	27	78	180
Standard deviation	5.07	5.78	5.52
Minimum	20	64	171
Maximum	37	91	194

**Table 2:** Descriptive data of study sample, showing average values of all isokinetic and unilateral countermovement jump variables normalized by body weight.

	Mean	Standard Error Mean	Median	Stand Dev	Min	Max
PT.Ext.R (Con)	2.84	0.08	2.90	0.46	2.04	3.83
PT.Ext.L (Con)	2.77	0.06	2.78	0.36	2.19	3.45
PT.Ext.R (Ecc)	3.53	0.14	3.49	0.79	2.20	5.13
PT.Ext.L (Ecc)	3.40	0.12	3.29	0.66	2.19	4.58
R.Jump Height (Kinetic)	14.90	0.29	14.80	1.66	12.20	19.40
L.Jump Height (Kinetic)	15.50	0.33	15.20	1.85	12.10	18.70
R.Force	1.87	0.02	1.85	0.14	1.60	2.13
L.Force	1.98	0.03	1.99	0.16	1.65	2.25
R.Power	2.87	0.07	2.98	0.39	2.21	3.52
L.Power	3.16	0.06	3.23	0.35	2.63	3.68

Note: Peak Torque of Extensor (PT.Ext); Dominant Side (R); Non-dominant Side (L); Concentric Contraction (Con); Eccentric Contraction (Ecc); Peak Torque in Nm/kg; Jump Height in cm; Force in N/kg; Power in Watts/kg.

**Table 3:** Person’s correlation matrix between all isokinetic and force platform variables measured from the countermovement jump of the dominant limb.

		PT.Ext.R (Con)	PT.Ext.R (Ecc)	PT.Flx.R (Con)	PT.Flx.R (Ecc)	R.Jump Height (Kinetic)	R.Jump Height (Time)	R.Force	R.Power
PT.Ext.R.Con	Pearson’s r	—							
	*p*-value	—							
	95% CI Upper	—							
	95% CI Lower	—							
PT.Ext.R.Ecc	Pearson’s r	0.80	—						
	*p*-value	<0.001 *	—						
	95% CI Upper	0.90	—						
	95% CI Lower	0.63	—						
PT.Flx.R.Con	Pearson’s r	0.53	0.45	—					
	*p*-value	0.00 *	0.01 *	—					
	95% CI Upper	0.74	0.69	—					
	95% CI Lower	0.22	0.13	—					
PT.Flx.R.Ecc	Pearson’s r	0.73	0.79	0.68	—				
	*p*-value	<0.001 *	<0.001 *	<0.001 *	—				
	95% CI Upper	0.86	0.90	0.83	—				
	95% CI Lower	0.52	0.62	0.44	—				
R.Jump.Height (Kinetic)	Pearson’s r	0.72	0.55	0.32	0.49	—			
	*p*-value	<0.001 *	<0.001 *	0.08 *	0.00 *	—			
	95% CI Upper	0.86	0.76	0.60	0.72	—			
	95% CI Lower	0.51	0.26	−0.04	0.17	—			
R.Force	Pearson’s r	0.00	0.03	−0.15	0.02	0.12	−0.21	—	
	*p*-value	1.00	0.87	0.42	0.92	0.52	0.25	—	
	95% CI Upper	0.35	0.38	0.21	0.36	0.45	0.15	—	
	95% CI Lower	−0.35	−0.32	−0.47	−0.33	−0.24	−0.52	—	
R.Power	Pearson’s r	0.22	0.19	−0.04	0.23	0.30	−0.02	0.75	—
	*p*-value	0.24	0.31	0.84	0.21	0.10	0.90	<0.001 *	—
	95% CI Upper	0.52	0.50	0.32	0.53	0.59	0.33	0.87	—
	95% CI Lower	−0.15	−0.17	−0.38	-0.13	-0.06	−0.37	0.55	—

Note. * (*p* < 0.05); Peak Torque in Nm/kg; Jump Height in cm; Force in N/kg; Power in Watts/kg.

**Table 4:** Person’s correlation matrix between all isokinetic and force platform variables measured from the countermovement jump of the non-dominant side.

		PT.Ext.L (Con)	PT.Ext.L (Ecc)	PT.Flx.L (Con)	PT.Flx.L (Ecc)	L.Jump Height (Kinetic)	L.Jump Height (Time)	L.Force	L.Power
PT.Ext.L (Con)	Pearson’s r	—							
	*p*-value	—							
	95% CI Upper	—							
	95% CI Lower	—							
PT.Ext.L (Ecc)	Pearson’s r	0.70	—						
	*p*-value	<0.001 *	—						
	95% CI Upper	0.84	—						
	95% CI Lower	0.47	—						
PT.Flx.L (Con)	Pearson’s r	0.45	0.52	—					
	*p*-value	0.01 *	0.00 *	—					
	95% CI Upper	0.69	0.73	—					
	95% CI Lower	0.12	0.20	—					
PT.Flx.L (Ecc)	Pearson’s r	0.48	0.68	0.72	—				
	*p*-value	0.01 *	<0.001 *	<0.001 *	—				
	95% CI Upper	0.71	0.83	0.86	—				
	95% CI Lower	0.16	0.43	0.50	—				
L.Jump.Height Height (Kinetic)	Pearson’s r	0.59	0.42	0.43	0.46	—			
	*p*-value	<0.001 *	0.02 *	0.01 *	0.01 *	—			
	95% CI Upper	0.78	0.67	0.68	0.69	—			
	95% CI Lower	0.31	0.09	0.10	0.13	—			
L.Force	Pearson’s r	−0.33	−0.48	0.08	−0.04	0.03	0.58	—	
	*p*-value	0.06	0.01	0.67	0.81	0.86	<0.001 *	—	
	95% CI Upper	0.02	−0.15	0.42	0.31	0.38	0.77	—	
	95% CI Lower	−0.61	−0.71	−0.28	−0.39	−0.32	0.29	—	
L.Power	Pearson’s r	−0.01	−0.15	0.27	0.37	0.19	0.58	0.64	—
	*p*-value	0.97	0.42	0.13	0.04	0.30	<0.001 *	<0.001 *	—
	95% CI Upper	0.34	0.21	0.57	0.64	0.50	0.77	0.81	—
	95% CI Lower	−0.35	−0.47	−0.09	0.02	−0.17	0.28	0.37	—

Note. * (*p* < 0.05); Peak Torque in Nm/kg; Jump Height in cm; Force in N/kg; Power in Watts/kg.

**Table 5:** Linear regression of right Side (based on PT.Ext.L.Con).

Predictor	Estimate	SE	Lower–Upper 95% CI		t	*p*
Intercept	7.65	1.62	4.33	10.98	4.73	<0.001
R.Jump.Height.Kinetic	2.97	0.84	1.25	4.69	3.54	0.001 *
**Model Fit Measures**			Overall Model Test			
Model	R	R^2^	F	df1	df2	*p*
1.00	0.726	0.527	40.70	1.00	30.00	<0.001

* statistically significant correlation.

**Table 6:** Linear regression of non-dominant side (based on PT.Ext.L.Con).

Predictor	Estimate	SE	Lower–Upper 95% CI		t	*p*
Intercept	6.57	2.32	1.82	11.328	2.84	0.01
L.Jump.Height.Kinetic	2.88	1.08	0.66	5.089	2.66	0.013 *
**Model Fit Measures**			Overall Model Test			
Model	R	R^2^	F	df1	df2	*p*
1	0.638	0.415	4.62	4	27	0.006

* statistically significant correlation.

## Data Availability

Additional data supporting reported results can be accessed by contacting the main author of the study.
